# Intradural extramedullary venous hemangioma mimicking meningioma: a case report and literature review

**DOI:** 10.1186/s12883-026-05272-y

**Published:** 2026-08-14

**Authors:** Lutong Wang, Xiaofang Wen, Shixing Zeng, Zhengran Yu, Yongyu Ye, Sharvesh Raj Seeruttun, Honglin Gu

**Affiliations:** 1 Department of Spine Surgery, Guangdong Provincial People’s Hospital (Guangdong Academy of Medical Sciences), Southern Medical University, Guangzhou, 510000 China; 2 Department of Orthopaedics, Guangdong Provincial People’s Hospital (Guangdong Academy of Medical Sciences), Southern Medical University, Guangzhou, 510000 China; 3 Department of Gastrointestinal Surgery, Department of General Surgery, Guangdong Provincial People’s Hospital (Guangdong Academy of Medical Sciences, Southern Medical University, Guangzhou, Guangdong 510080 China

**Keywords:** Intradural extramedullary tumor, Venous hemangioma, Spinal canal lesion, Magnetic resonance imaging, Microsurgical resection

## Abstract

**Background:**

Intradural extramedullary hemangiomas of the spinal canal are rare, and the venous subtype is particularly uncommon. Their imaging appearance can overlap with more frequent intradural extramedullary tumors, which increases the risk of misdiagnosis. This case report aims to describe an intradural extramedullary venous hemangioma that mimicked meningioma on MRI and to highlight key surgical considerations.

**Case presentation:**

A 49-year-old man presented with a five-month history of progressive numbness, radicular pain, and weakness in the left lower limb, which progressed to gait disturbance. Neurological examination revealed hypoesthesia over the lateral left leg and dorsum of the foot, with motor weakness of the extensor hallucis longus and hamstring muscles (MRC grade 3/5). Magnetic resonance imaging demonstrated an 11 × 9 mm intradural extramedullary lesion at T12/L1 that appeared slightly hyperintense on T1-weighted images, isointense on T2-weighted images, and showed mild homogeneous enhancement with a subtle dural tail sign, suggesting a diagnosis of meningioma, following which the patient underwent posterior T12 total laminectomy with lamina replantation and gross total resection was achieved without nerve root sacrifice. Microsurgical excision revealed a dark red, mulberry-like vascular mass densely adherent to the spinal cord and cauda equina nerve roots. Histopathological analysis demonstrated proliferated vascular channels of variable lumen size, positive endothelial staining for ERG, and a Ki-67 index of approximately 2%, confirming venous hemangioma. Postoperatively, the patient experienced immediate pain relief and progressive neurological improvement. At one-year follow-up, motor strength had fully recovered, with only mild residual numbness and no radiological evidence of recurrence.

**Conclusion:**

This case illustrates a rare intradural extramedullary venous hemangioma that radiologically mimicked meningioma. Complete microsurgical resection provided promising neurological recovery and highlights the importance of considering vascular tumors in the differential diagnosis of intradural spinal lesions.

**Supplementary Information:**

The online version contains supplementary material available at https://doi.org/10.1186/s12883-026-05272-y.

## Introduction

Spinal hemangiomas are benign vascular lesions resulting from abnormal blood vessel proliferation. Most hemangiomas occur within the vertebral bodies and are frequently incidental findings. In contrast, hemangiomas arising within the spinal canal are uncommon and have been reported to account for a small proportion (2–7%) of intraspinal tumors [1]. Hemangiomas are commonly classified as capillary, cavernous, arteriovenous, and venous subtypes [2], with capillary and cavernous hemangiomas most often described in published reports. Intradural extramedullary venous hemangiomas are particularly rare, and due to the limited number of published cases, their imaging features and optimal surgical strategy are not well defined.

The clinical presentation of hemangiomas is typically related to mass effect on the spinal cord or cauda equina, ranging from radicular pain and sensory disturbance to progressive weakness, and occasionally acute neurological decline. Magnetic resonance imaging (MRI) is the preferred modality for assessment; however, its imaging characteristics are highly variable [3, 4], as they may overlap with more common intradural extramedullary tumors such as meningioma or schwannoma, which can lead to preoperative misdiagnosis. Surgical resection remains the mainstay of treatment for hemangiomas, with complete excision generally resulting in favorable neurological outcomes and low recurrence rates. However, surgery remains challenging as hemangiomas are often highly vascular and densely adherent to the spinal cord or nerve roots, increasing bleeding risk and the likelihood of neural injury [5]. In this regard, preoperative embolization may be considered for larger or more vascular lesions, but its role is limited in small intradural tumors [6].

Here, we present a case of an intradural extramedullary venous hemangioma at T12/L1 in a 49-year-old man, which was initially suspected to be meningioma on MRI. This case aims to emphasize venous hemangioma as a differential diagnosis for intradural extramedullary tumors, and summarize practical intraoperative considerations that support safe gross total resection and favorable outcome.

## Case presentation

A 49-year-old man presented with a five-month history of progressive numbness, radicular pain, and weakness in the left lower limb, which gradually led to gait disturbance. He denied bowel or bladder dysfunction. His past medical history included grade 2 hypertension and type 2 diabetes mellitus. He denied fever, weight loss, recent trauma, and prior malignancy.

On admission, neurological examination revealed hypoesthesia over the lateral aspect of the left leg and the dorsum of the foot. Motor testing demonstrated weakness of the left extensor hallucis longus and hamstring muscles, with muscle strength graded 3/5 on the Medical Research Council (MRC) scale. Muscle tone was normal, deep tendon reflexes at the knee and ankle were preserved, and no pathological reflexes were elicited. Patient-reported outcome measures included a Visual Analog Scale (VAS) score of 4/10 for lumbar pain and 5/10 for radicular pain, an Oswestry Disability Index (ODI) score of 50% (severe disability), and a Short Form-12 (SF-12) score of 36 (impaired quality of life). Given the T12/L1 intradural location, the unilateral distal leg symptoms were considered compatible with localized compression of the descending cauda equina nerve roots, rather than a true conus medullaris syndrome.

**Fig. 1:**
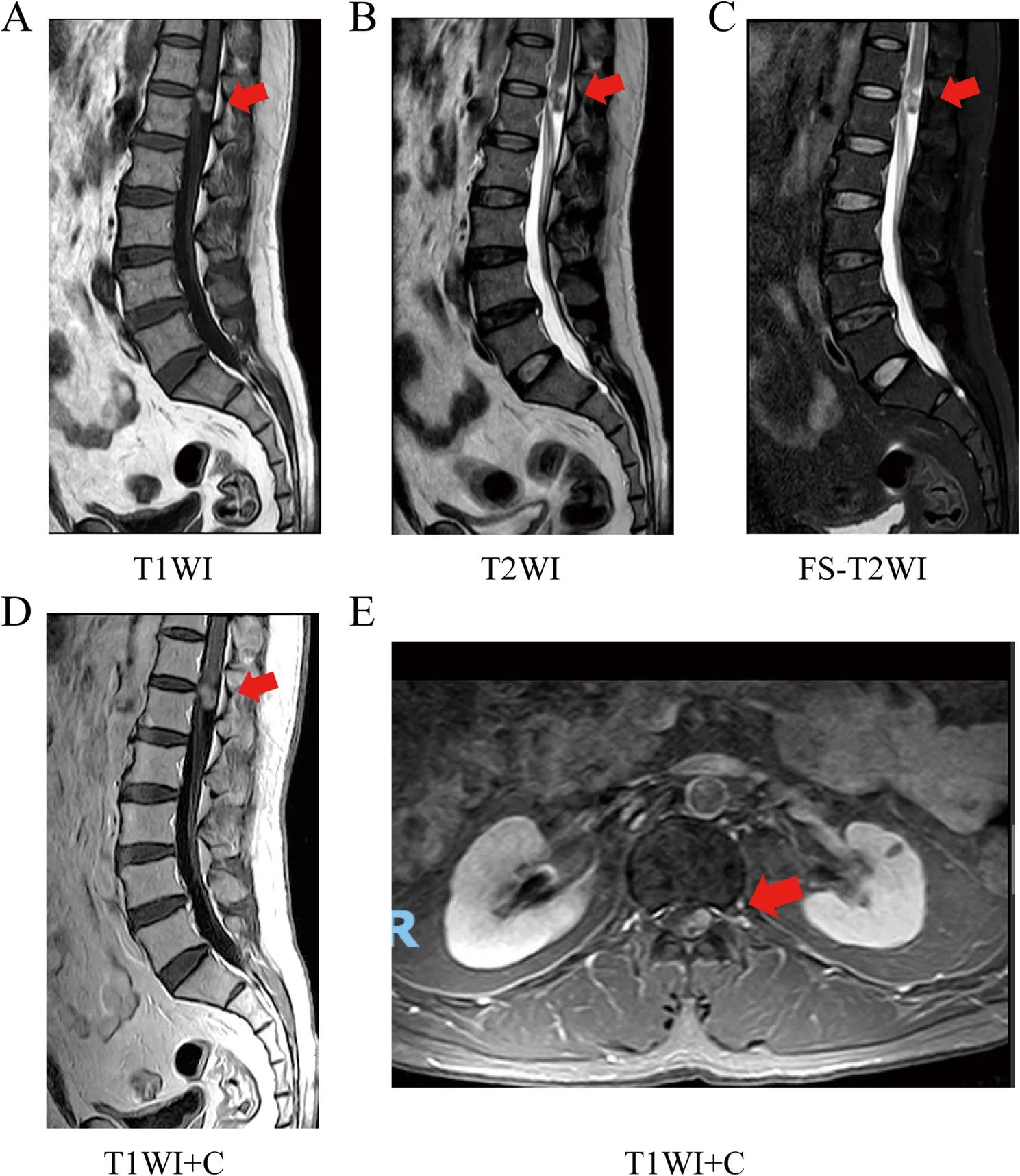
Preoperative radiological findings of intradural extramedullary venous hemangioma. **A** Sagittal T1-weighted MRI showing a slightly hyperintense, well-circumscribed intradural extramedullary lesion at the T12/L1 level. **B** Sagittal T2-weighted MRI demonstrating isointense signal characteristics. **C** Sagittal T2 fat-suppressed MRI confirming the lesion without associated marrow edema or vertebral destruction. **D** Sagittal T1-weighted post-contrast MRI showing mild homogeneous enhancement with a subtle dural tail sign (arrow), radiologically suggestive of meningioma. **E** Axial T1-weighted post-contrast MRI at the T12/L1 level demonstrating the lesion compressing the spinal cord.

Routine laboratory investigations, including complete blood count, liver and renal function tests, and inflammatory markers, were within normal limits. As an adjunctive component of our institutional preoperative protocol, standing plain radiography of the lumbar spine was obtained to assess baseline spinal alignment, which demonstrated normal sequence without pre-existing instability. Computed tomography (CT) showed no evidence of bony destruction. Magnetic resonance imaging (MRI) revealed an 11 × 9 mm well-circumscribed intradural extramedullary lesion at the T12/L1 level. The lesion appeared slightly hyperintense on T1-weighted images, isointense on T2-weighted images, and showed mild homogeneous enhancement following gadolinium administration (Fig. 1). A subtle dural tail sign was observed, favoring a radiological diagnosis of meningioma. The main differential diagnoses included schwannoma and other vascular lesions; an intradural extramedullary hemangioma was considered but was regarded as less likely preoperatively. Preoperative spinal angiography was not performed because the primary preoperative differential diagnosis based on MRI—particularly given the dural tail sign—was a solid tumor such as a meningioma or schwannoma, rather than a high-flow vascular malformation (e.g., arteriovenous malformation or fistula) that would necessitate vascular evaluation. Furthermore, the patient’s progressively worsening neurological deficits indicated the need for prompt surgical decompression.

**Fig. 2:**
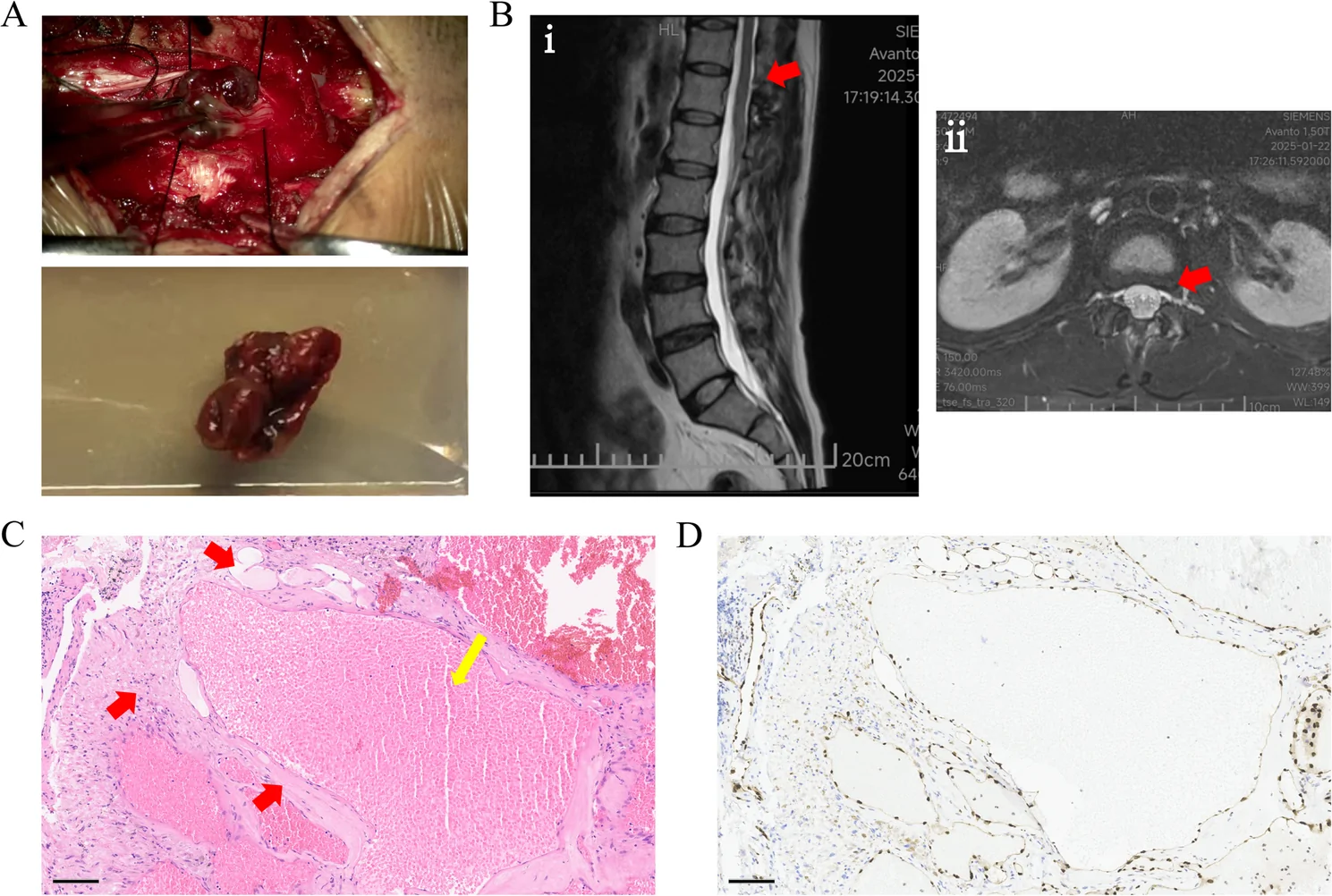
Intraoperative and pathological features with postoperative outcome. **A** Intraoperative photograph showing a dark red, mulberry-like vascular mass adherent to the spinal cord and cauda equina nerve roots. **B** Postoperative sagittal T2-weighted (i) and axial T2-weighted (ii) MRI at one year confirming complete spinal cord decompression without residual or recurrent tumor. **C** Hematoxylin and eosin (H&E) staining (×200) demonstrating proliferated vascular channels with irregular wall thickness and variable lumen size, consistent with venous hemangioma. (red arrow: saccular dilated blood vessel with multiple layers of smooth muscle cells on the sac wall, yellow arrow: blood clot; Scale bar: 100 μm). **D** Immunohistochemistry for ERG (×200) highlighting the vascular endothelial nature of the lesion. Scale bar: 100 μm

On August 30, 2023, the patient underwent posterior T12 total laminectomy with lamina preservation for replantation. Continuous intraoperative neurophysiological monitoring (IONM), including both motor evoked potentials (MEPs) and somatosensory evoked potentials (SSEPs), was utilized to ensure neural safety. Microsurgical tumor excision was performed under high magnification. After dural opening, a dark red, mulberry-like vascular lesion measuring approximately 10 × 8 mm was identified. The lesion was densely adherent to the spinal cord and cauda equina nerve roots and was highly vascular, requiring meticulous bipolar coagulation and careful dissection. Gross total resection was achieved without sacrificing nerve roots. Throughout the resection process, the MEP and SSEP signals remained stable without any significant amplitude decrement or latency delay, confirming the functional preservation of the neural structures (supplementary file, Figure S1). The estimated blood loss was 200 mL, and no transfusion was required. The dura was closed, the T12 lamina was repositioned to maintain spinal stability, and no intraoperative complications occurred (Fig. 2A).

Histopathological examination revealed proliferated vascular channels with irregular wall thickness and variably sized lumina, surrounded by axonal and glial tissue. Immunohistochemistry demonstrated positivity for Vimentin (+++), D2-40 (++–+++), glial fibrillary acidic protein (GFAP) (glial +), Olig2 (plasma cells +), S100 (axonal +), SOX10 (axonal +), protein gene product 9.5 (PGP9.5) (axonal +), neurofilament (NF) (axonal +), O6-methylguanine-DNA methyltransferase (MGMT) (partial +++), and ETS-related gene (ERG) (vascular +). In contrast, Cytokeratin (CK), epithelial membrane antigen (EMA), progesterone receptor (PR), and somatostatin receptor 2 (SSTR2) were negative, arguing against epithelial or meningioma-related differentiation. The Ki-67 proliferation index was approximately 2% in hotspot regions, supporting a low proliferative activity. Reticulin staining was negative. Taken together, these pathological and immunophenotypic findings confirmed the diagnosis of an extramedullary intradural venous hemangioma (Fig. 2B–C). These findings effectively differentiated our lesion from cavernous hemangiomas, which lack organized venous walls, and from venous malformations, which lack the proliferative endothelial characteristics confirmed here by ERG positivity and specific histological growth patterns.

The postoperative recovery was uneventful. The patient reported immediate relief of radicular pain and did not develop new neurological deficits. He was mobilized early, and no postoperative spinal instability was observed. At six-month telephone follow-up, he reported only mild residual numbness, with near-complete recovery of motor strength. At one-year follow-up, muscle strength had fully recovered to grade 5/5, and repeat MRI confirmed complete decompression of the spinal cord with no residual or recurrent tumor. No adjuvant therapy was required.

## Discussion

This case highlights the diagnostic uncertainty and surgical challenges posed by intradural extramedullary venous hemangiomas. These benign vascular lesions are rare within the spinal canal and are therefore frequently misdiagnosed preoperatively. While capillary and cavernous intradural extramedullary hemangiomas have been reported sporadically, venous hemangiomas remain exceptionally uncommon. Recent case reports and reviews indicate that these lesions can closely resemble more common intradural tumors, particularly meningioma and schwannoma, on MRI [5]. A summary of published cases of intradural extramedullary venous hemangiomas is provided in Table 1.

**Table 1:** Summary of previously reported intradural extramedullary venous hemangiomas

Author/year	Number of Cases	Age (years)/ sex	Duration of illness	Clinical Presentation	Tumor location	Spinal Level	MRI findings			Cystic/nerve root attachment	Treatment and outcome	Ref.
							T1-Weighted Image	T2-Weighted Image	Contrast Enhancement			
Nishimura/2013	1	54/F	4 years; worsening 2 weeks	Low-back pain; left lower limb pain and numbness	IDEM	T12–L1	Slight hyper	Iso	No	+/+	CSR; good recovery at 24 months	[7]
Present case	1	49/M	5 months	left lower limb pain, numbness and weakness	IDEM	T12–L1	Hyper	Iso/hypo	Heterogenous	+/+	CSR; good recovery at 12 months	

*CSR* Complete surgical removal, *IDEM* Intradural extramedullary

Clinically, our patient developed progressive radicular pain, sensorimotor deficits, and gait disturbance, consistent with nerve root or cauda equina compression. This pattern is broadly consistent with prior reports of intradural extramedullary hemangiomas. For example, one conus medullaris case showed cyst-like features that mimicked the margins of hemangioblastoma and delayed the correct diagnosis [8]. Although acute neurological deterioration can occur due to intralesional hemorrhage, the gradual progression in our patient suggested slow enlargement rather than an acute bleeding event.

Radiologically, the diagnostic challenge was substantial. MRI demonstrated mild homogeneous enhancement with an apparent dural tail sign, leading to a preoperative diagnosis of meningioma. However, accumulating evidence shows that the dural tail sign is not specific and may also be seen in vascular tumors, including capillary hemangiomas [9]. Additionally, a report on spinal capillary hemangiomas described multiple cases in which MRI findings resulted in incorrect preoperative diagnoses such as schwannoma or meningioma [5], which supports including hemangioma in the differential diagnosis of enhancing intradural extramedullary lesions, particularly when imaging findings are not fully typical of a single entity. In our case, the subtle dural tail sign on post-contrast MRI (Fig. 1D) was misleading, leading to a preoperative suspicion of meningioma. This underscores that the dural tail sign is not pathognomonic for meningioma and can be seen in vascular lesions.

From an anatomical perspective, the lesion presented a favorable surgical scenario, as its dorsal location allowed immediate visualization upon dural opening without requiring significant retraction of the conus medullaris. However, the true surgical challenge lay in the tumor’s distinct tissue characteristics; unlike the firm, encapsulated nature of typical meningiomas or schwannomas, this venous hemangioma presented as a soft, friable, ‘mulberry-like’ mass with high vascularity. The lesion lacked a distinct capsule and was densely adherent to the pia mater and cauda equina nerve roots, intertwining with these delicate structures. Consequently, maintaining a bloodless field through meticulous micro-dissection and controlled bipolar coagulation was essential to prevent neural injury. Furthermore, we were unable to pinpoint a precise site of origin; the lesion’s dense adhesion to both pia mater and nerve roots—corroborated by the presence of axonal and glial tissues on histopathology—suggests that it may not arise from a single anatomical structure, but rather exhibits a propensity to involve adjacent neurovascular elements through reactive changes [5].

Long-term spinal stability is an important consideration in surgical planning. In this case, we performed laminoplasty (lamina replantation) rather than a conventional laminectomy to preserve the posterior spinal elements. Recent studies support this approach, as laminoplasty has been shown to reduce postoperative deformity and better maintain spinal alignment compared with laminectomy. The patient’s favorable outcome, with resolution of radicular pain, full motor recovery at one year, and no recurrence on follow-up MRI, may be partly attributed to the combination of complete tumor resection and preservation of the posterior spinal architecture.

Synthesizing our experience with previously reported cases, we propose the following surgical pearls for managing intradural extramedullary venous hemangiomas:

1. Early Vascular Control: Prioritize the identification and coagulation of feeding vessels on the tumor surface early in the dissection to minimize intraoperative bleeding.
2. Microsurgical Precision: Given the dense adhesion to pia mater and nerve roots, blunt dissection should be strictly avoided. Instead, high-magnification micro-dissection combined with low-power, controlled bipolar coagulation is essential to separate the tumor from delicate neural fascicles.
3. Management of Origin Ambiguity: Surgeons should be prepared for the absence of a distinct tumor capsule or clear origin. Radical resection must be carefully balanced against the risk of sacrificing function-critical nerve roots.
4. Spinal Structural Preservation: We advocate for laminoplasty (lamina replantation) rather than conventional laminectomy, particularly in the thoracolumbar junction, to prevent long-term iatrogenic spinal deformity and maintain alignment.

Histopathological confirmation remains essential for definitive diagnosis. Immunohistochemical analysis positive for endothelial markers (e.g., ERG) and negative for meningioma markers (e.g., EMA) confirmed the venous hemangioma diagnosis. This aligns with current literature emphasizing the necessity of histological evaluation to differentiate vascular tumors from more common intradural pathologies when imaging is ambiguous [10]. Furthermore, diagnostic accuracy is often confounded by terminological inconsistencies in the literature, where ‘angioma’ and ‘hemangioma’ are frequently used interchangeably. According to current World Health Organization (WHO) classifications, it is imperative to distinguish true venous hemangiomas—which are characterized by active, albeit benign, endothelial proliferation (as evidenced by our Ki-67 and ERG findings)—from venous malformations or angiomas, which are primarily congenital structural anomalies lacking active proliferative characteristics.

Applying this diagnostic rigor to the existing literature, it is crucial to establish strict criteria when comparing these rare vascular lesions. Maintaining rigorous pathological and anatomical distinctions is vital for accurate surgical planning and prognostic evaluation. Consequently, Table 1 has been meticulously curated to include only confirmed cases of intradural extramedullary (IDEM) venous hemangiomas. While several other spinal vascular lesions have been reported, they present fundamentally different clinical and surgical paradigms. For instance, epidural venous hemangiomas, including those presenting with acute hemorrhage following epidural analgesia [11], dictate a different surgical urgency and approach compared to IDEM tumors. Similarly, cases with mixed histology, such as pregnancy-related capillary-cavernous hemangiomas [12], represent distinct pathological entities.

This case contributes additional evidence regarding a rare spinal lesion. First, it shows that imaging findings usually considered typical for meningioma, such as the dural tail sign, can also occur in vascular tumors and lead to misdiagnosis. Second, it demonstrates that careful microsurgical resection can allow safe and complete removal of a lesion that is both highly vascular and adherent to nerve roots, with good neurological recovery. Lastly, by adding to the published report of venous hemangiomas, this case helps to expand current knowledge of their clinical presentation, imaging characteristics, and pathology, and highlights the need for long-term follow-up to better understand their natural history.

## Conclusion

In conclusion, intradural extramedullary venous hemangiomas are rare lesions and may be misdiagnosed preoperatively, posing difficulties during resection because of their vascularity and adhesion to neural structures. Careful preoperative assessment, meticulous microsurgical resection with attention to preserving spinal stability, and histopathological confirmation are essential for safe and effective management. By reporting such cases, clinicians can improve awareness, expand the spectrum of differential diagnoses, and provide guidance for optimal surgical treatment of this rare entity.

## Supplementary Information

Supplementary Material 1.

## Data Availability

All data generated or analysed during this study are included in this published article and its supplementary information files.

## Abbreviations

MRI: Magnetic resonance imaging
MRC: Medical Research Council
VAS: Visual Analog Scale
ODI: Oswestry Disability Index (ODI)
SF-12: Short Form-12 (SF-12)
CT: Computed Tomography (CT)
CSR: Complete surgical removal
IDEM: Intradural extramedullary
