# Supplementary material for: Intradural extramedullary venous hemangioma mimicking meningioma: a case report and literature review
Source: BMC Neurol. 2026 Aug 14;26:660. doi: 10.1186/s12883-026-05272-y (PMC13681784; doi:10.1186/s12883-026-05272-y)
Supplement: Supplementary file 1 [file 12883_2026_5272_MOESM1_ESM.docx]

**Intradural Extramedullary Venous Hemangioma Mimicking Meningioma: A Case Report and Literature Review**

Lutong Wang ^a,1^, Xiaofang Wen ^b, 1^, Shixing Zeng ^a^, Zhengran Yu ^a^, Yongyu Ye ^a^, Sharvesh Raj Seeruttu ^c, *^, Honglin Gu ^a, *^

^a^ Department of Spine Surgery, Guangdong Provincial People's Hospital (Guangdong Academy of Medical Sciences), Southern Medical University, Guangzhou, 510000, China.

^b^ Department of Orthopaedics, Guangdong Provincial People's Hospital (Guangdong Academy of Medical Sciences), Southern Medical University, Guangzhou, 510000, China.

^c^ Department of Gastrointestinal Surgery, Department of General Surgery, Guangdong Provincial People's Hospital (Guangdong Academy of Medical Sciences), Southern Medical University, Guangzhou 510080, Guangdong, China

*Corresponding author: Sharvesh Raj Seerutt, cancer.surgery.phd@gmail.com; Honglin Gu, guhonglin@gdph.org.cn.

^1^ Lutong Wang and Xiaofang Wen contributed equally.

Figure S1. The representative screenshots demonstrating the stable intraoperative MEP and SSEP recordings.
